# Hepatic Segrnentectomy on Primary Liver Cancer with Situs Inversus Totalis

**DOI:** 10.1155/1996/29894

**Published:** 1996

**Authors:** W. Kamiike, T. Itakura, H. Tanaka, N. Hatanaka, M. Nakamuro, M. Miyata, H. Izumi

**Affiliations:** 1 Department of Surgery National Federation of Health Insurance Osaka Central Hospital Osaka Japan; 2 The First Department of Surgery Osaka University Medical School 2-2 Yamada-oka Suita 565 Japan

## Abstract

We present the first case treated by hepatic segmentectomy in a 69-year-old woman with primary liver cancer and situs inversus totalis. The situs inversus did not cause any technical problems during the operation, which was conducted under guidance *of* intraoperative ultrasonography.


*HPB Surgery*, 1996, Vol.9, pp.169–173
Reprints available directly from the publisher
Photocopying permitted by license only

© 1996 OPA (Overseas Publishers Association)
Amsterdam B.V. Published in The Netherlands
by Harwood Academic Publishers GmbH
Printed in Malaysia

CASE REPORT

### Hepatic Segmentectomy on Primary Liver Cancer with Situs Inversus Totalis

W. KAMIIKE[2], T. ITAKURA[1], H. TANAKA[1], N. HATANAKA[2],
M. NAKAMURO[2], M. MIYATA[2] and H. IZUMI[1].

[1]Department of Surgery, National Federation of Health Insurance Osaka Central Hospital, Osaka, Japan

Visiting surgeon[2]. Parent institution: The First Department of Surgery,
Osaka University Medical School

*(Received 1 May 1994)*

We present the first case treated by hepatic segmentectomy in a 69-year-old woman with primary liver cancer and situs inversus totalis. The situs inversus did not cause any technical problems during the operation, which was conducted under guidance *of* intraoperative ultrasonography.

KEY WORDS: Primary liver cancer situs inversus hepatic segmentectomy intraoperative sonography

### INTRODUCTION

Situs inversus totalis is a rare congenital condition, occurring with an incidence of 1:5000 to 1:10000 adults[1], in which there is mirror-image transposition of both the abdominal and thoracic viscera. This condition, which has the potential to cause problems during operation, was simplified by adequate preoperative examination and intraoperative ultrasonography. We successfully performed resection of the postero-superior segment of the liver. This is the first reported case of hepatic segmentectomy performed on primary liver cancer complicated with transposition of the viscera.

### CASE REPORT

A 69-year-old Japanese woman who had a history of liver cirrhosis was admitted to the National Federation of Health Insurance Osaka Central Hospital on June 21, 1991 because of a high plasma level of alphafetoprotein (AFP). At the age of 20 years, she had undergone laparotomy for bowel obstruction, and situs inversus totalis was detected at that time. The physical examination on admission revealed that normal heart sounds were audible in the right side of the chest. The liver and spleen were not palpable. There was no ascites. Her biochemical profile was remarkable, because plasma AFP was elevated to 291 ng/ml, serum bile acid was elevated to 51 μmol/l, and indocyanine green retention at 15 minutes was 19.3%. Serum alanine aminotransferase (ALT), serum aspartate aminotransferase (AST), serum bilirubin and total protein were all within their normal limits.

Chest x-rays obtained on admission demonstrated dextrocardia. Abdominal ultrasonography revealed situs inversus totalis; that is, the liver and inferior vena cava were on the left side, and showed a hypodense mass in the postero-superior segment *(Seg VII)*, according to Couinaud's classification of the liver segments, which was about 3 cm in diameter (Fig. 1). An abdominal computed tomographic scan showed a low-density area in the postero-superior segment of the liver. Selective celiac arteriography revealed a faint tumor stain supplied by what would have been the right hepatic artery in usual cases (Fig. 2). These findings were consistent with a diagnosis of primary liver cancer in the postero-superior segment.

*Correspondence to*: The First Department of Surgery, Osaka University Medical School, 2–2 Yamada-oka, Suita 565, Japan. TEL; 06-879-3153, FAX; 06-879-3159

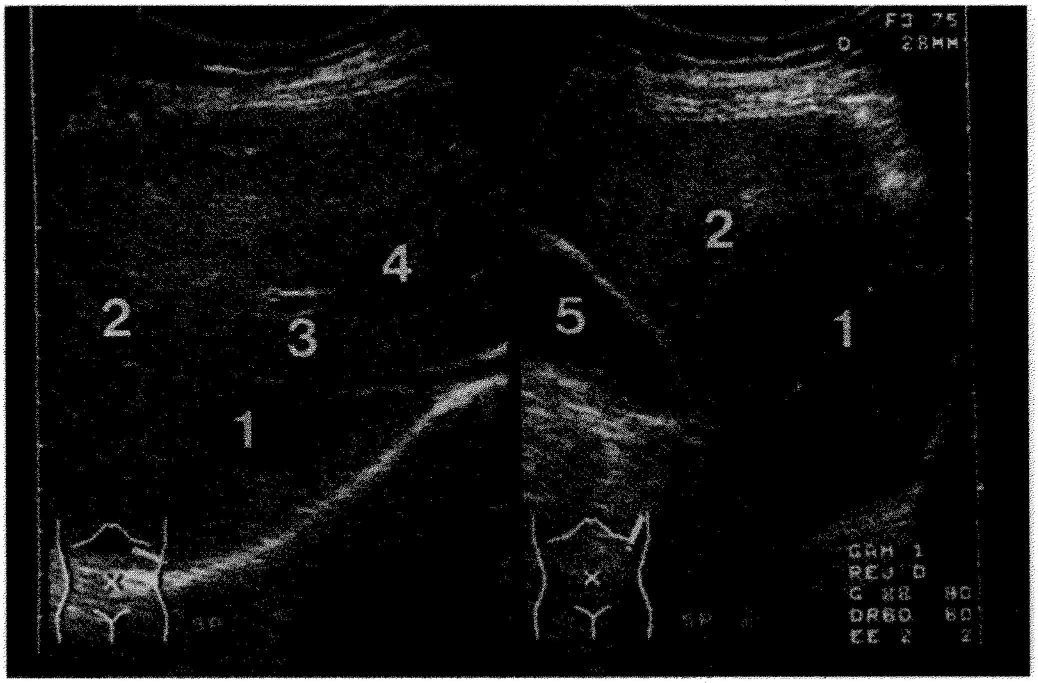

**Figure 1** Preoperative ultrasonogram shows a mirror image with rightsided structures on the left. A mass is demonstrated on two different scanning planes at Right angles to each other. Left subcostal (A) and intercostal scan (B). 1: tumor; 2: right hepatic vein in usual cases; 3: portal vein of the anterior segment; 4: middle hepatic vein; 5: gall bladder.

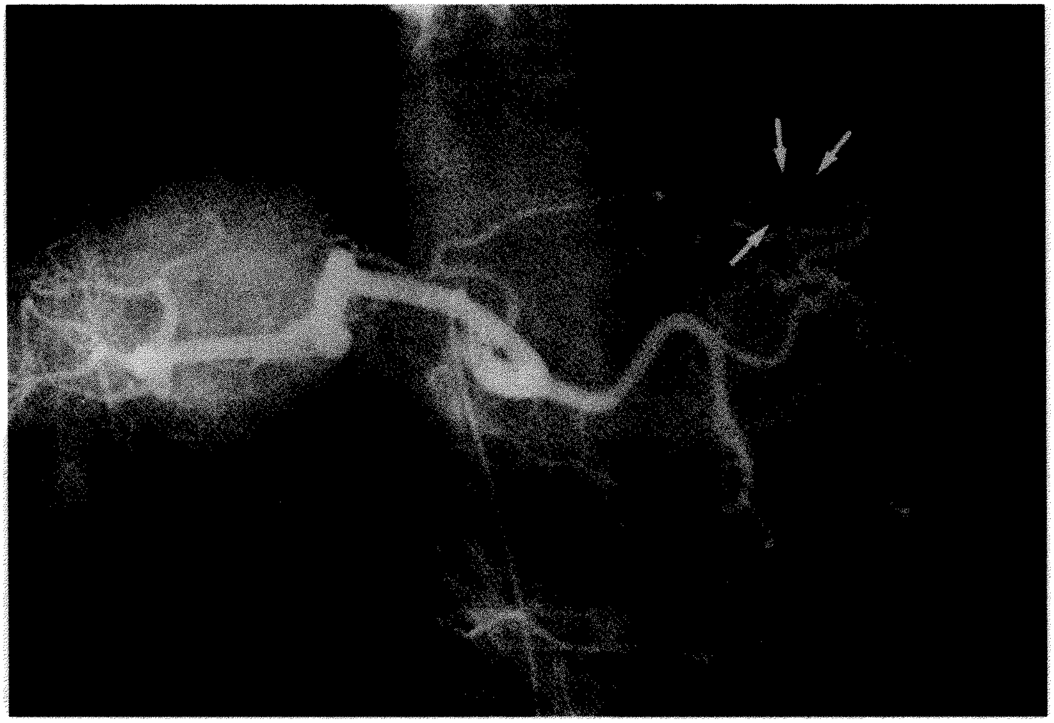

**Figure 2** A selective celiac angiogram. The left hepatic artery is apart from what would be the left gastric artery in usual cses, otherwise it is normal apart from reversal of the celiac vasculature is shown. The arrow indicates a faint tumor stain.

Then 6 ml of Lipiodol was injected via the right hepatic artery for transcatheter arterial embolization (TAE). Fourty days after the TAE, computed tomography showed more clearly a 20-mm tumor lesion (Fig. 3). The plasma AFP level had decreased to 94 ng/ml.

On August 6, 44 days after the TAE, the operation was performed. The diagnosis of situs inversus totalis was confirmed at laparotomy. The liver showed hepatic cirrhosis. Resection of the postero-superior segment was attempted. The right posterior pedicle was identified and clamped. Then minor resection of the postero-superior segment was performed using the technique of microwave coagulation (Microtaze; Nippon Shoji Kaisha, LTD.) and a Cavitron Ultrasonic Surgical Aspirator (CUSA; Valley Lab Inc.; Stanford, CT). Under guidance with intraoperative ultrasonography, the right hepatic vein in usual cases was preserved. The surgery was uncomplicated, with a blood loss of 1010 ml and an operating time of 5.5 hours. The resected tumor was well-encapsulated and 25 × 20 × 20 mm in size. Microscopically, the tumor was diagnosed as a combined hepatocellular and cholangio-cellular carcinoma. The liver showed inactive cirrhosis with moderate fatty change.

The post-operative course was uneventful, and the patient is doing well 32 months after the operation. The AFP concentration decreased to 46 ng/ml on the first postoperative day, and then gradually fell to 4 ng/ml one month after the operation.

### DISCUSSION

Situs inversus is a term used to describe left-to-right transposition of the normally asymmetrical organs of the body, and transposition of both the thoracic and abdominal organs is termed situs inversus totalis. This anomaly corresponds to a homologous region in humans located on the long arm of chromosome 12[2]. Recently, Yost suggested that left-right axial information is contained in the extracellular matrix early in development and is independently transmitted to the cardiac and visceral primordia[3]. Although this anomaly is not considered to be a premalignant entity, 13 cases of malignancy associated with situs inversus totalis have been reported[4–16]. Two of those 13 cases were primary liver cancer. In 1983, Kanematsu described the first case of a primary hepatocellular carcinoma

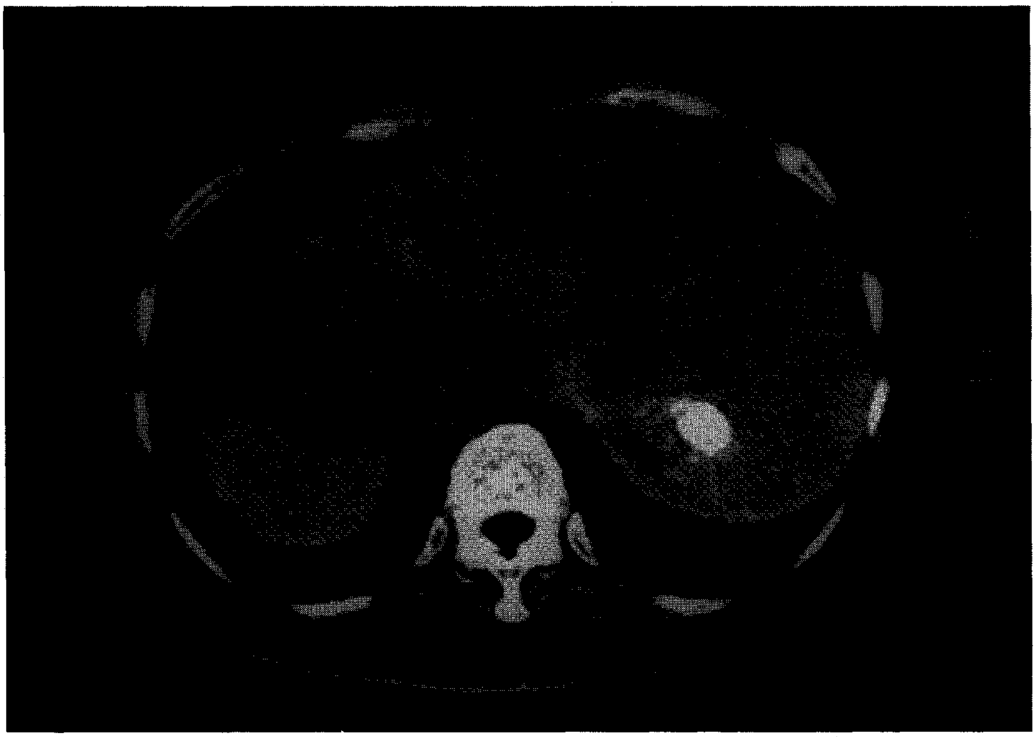

**Figure 3** Abdominal computed tomographic scan. Precontrast CT 40 days following the injection of Lipiodol into what would have been the right hepatic artery in usual cases shows an extremely hyperdense nodule in the postero-superior segment.

in a 37-year-old man with situs inversus totalis who underwent what would have been a right hepatic lobectomy in usual cases[10]. That patient died of recurrent neoplasm of the liver one year and four months after the lobectomy. In 1989, Kim et al. reported another case of a hepatocellular carcinoma in a 66-year-old woman with situs inversus totalis[15]. This patient developed hepatocellular carcinoma associated with stomach cancer, and she was successfully treated by right hepatic lobectomy in usual cases and distal gastrectomy. Although hepatitis B surface antigen was positive in both cases, the preoperative hepatic function was good enough to permit right hepatic lobectomy. However, our patient had suffered from liver cirrhosis. Some hepatocellular carcinoma patients who have cirrhosis show deterioration of hepatic function after hepatectomy, and some of them develop postoperative hepatic insufficiency[17]. According to the prognostic score for the operability of hepatectomy estimated by Yamanaka and Okamoto[18], we decided that lobectomy was impossible in our case. Thus, only the postero-superior segment was resected. Identification of the hepatic vessels, especially the right hepatic vein, is the most important point of this operation. We obtained a mirror image of the liver by using intraoperative ultrasonography to understand the anatomy of this patient. Resection of the postero-superior segment was performed easily and without complications due to our precise understanding of the patient's anatomy.

We would like to emphasize that, when operating on a patient with primary liver cancer and situs inversus totalis, precise preoperative anatomical evaluation is important, and intraoperative ultrasonography is highly useful.

### REFERENCES

1. Varano, N.R. and Merklin, R.J. (1960) Situs inversus: review of the literature, report of four cases and analysis of the clinical implications. *Journal of the International College of Surgeons*, **33** 131–148.
2. Brueckner, M., D'Eustachio, P. and Horwich, A.L. (1989) Linkage mapping of a mouse gene, iv, that controls left-right asymmetry of the heart and viscera. *Proceedings of the National Academy of Sciences*, **86** 5035–5038.
3. Yost, H.J. (1992) Regulation of vertebrate left-right asymmetries by extracellular matrix. *Nature*, **357** 158–161.
4. Allen, F.R.W.K. (1936) A case of malignant tumor of the stomach in a male with transposition of the viscera. *Indian Medical Gazette*, **71** 32.
5. Blegen, H.M. (1948) Surgery in situs inversus. *Annals of Surgery*, **129** 244–259.
6. Baruah, B.D. and Chari, M.V. (1952) Kartagener syndrome with bronchogenic carcinoma. *Journal of the Indian Medical Association*, **21** 438–439.
7. Shank, P.J. and Stafford, R.P. (1953) Complete situs inversus associated with cholelithiasis and complicated by carcinoma of the gallbladder. *Review of Gastroenterology*, **20** 167–173.
8. Thompson, J.R. (1963) Bronchogenic carcinoma complicating situs inversus totalis. *Diseases of the Chest*, **44** 317–319.
9. Write, C.B. and Morton, C.B.II (1971) Situs inversus totalis with adenocarcinoma of the cecum: Case report. *Annals of Surgery*, **37** 65–66.
10. Kanematsu, T., Matsumata, T., Kohno, H., Sugimachi, K. and Inokuchi, K. (1983) Hepatocellular carcinoma with situs inversus. *Cancer*, **51** 549–552.
11. Ibrahim, E.M., Al-Idrissi, H., Al-Faraq, A., Al-Tamimi, D. and Perry, W. (1984) Situs inversus totalis with embryonal cell carcinoma of ovaries. *Gynecologic Oncology*, **18** 270–273.
12. Talerman, A. (1984) A patient with situs inversus totalis and embryonal carcinoma of the ovary. *Gynecologic Oncology* **19** 371–372.
13. Abdel-Dayem, H.M., Motawi, S., Jahan, S. and Kubasik, H. (1984) Liver metastatis from embryonal carcinoma of the ovary with complete situs inversus: first reported case and review of literature. *Clinical Nuclear Medicine*, **9** 558–560.
14. Bertini, J. and Boileau, M.A. (1987) Renal cell carcinoma in a patient with situs inversus totalis. *Journal of Surgical Oncology*, **34** 29–31.
15. Kim, Y. II, Tada, I., Kuwabara, A. and Kobayashi, M. (1989) Double Cancer of the liver and stomach with situs inversus totalis: A case report. *Japanese Journal of Surgery*, **19** 756–759.
16. Organ, B.C., Skandalakis, L.J., Gray, S.W. and Skandalakis, J.E. (1991) Cancer of bile duct with situs inversus. *Archives of Surgery*, **126** 1150–1153..
17. Lin, T.Y. and Chen, C.C. (1979) Metabolic function and regeneration of cirrhotic and non-cirrhotic livers after hepatic lobectomy in men. *Annals of Surgery*, **190** 959–972.
18. Yamanaka, N., Okamoto, E., Kuwata, K. and Tanaka, N. (1984) A multiple regression equation for prediction of posthepatectomy liver failure. *Annals of Surgery*, **200** 658–663.

### COMMENTARY

Vertebrates are unique in that they have mirror symmetry for external structures but have left- or right-handed asymmetry for internal organs[1]. The question of how the visceral organs, which originate in the midline, consistently migrate to the normal right or left side during development in a non-random fashion, has intrigued anatomists for ages[2].

Situs inversus is rare in human beings and the incidence ranges between 1: 1,000 and 1: 10,000 depending on the population surveyed[3]. In situs inversus totalis, a right/left mirror image of transposition of the abdominal and thoracic viscerae occurs. In heterotaxy, the abdominal viscera may be inverted, whereas the thoracic contents may be normal, or vice versa. In isomerism, the body appears symmetric with bilateral left or right features[2]. The organs are often abnormal in each of these syndromes. The liver may be bilobed or symmetric, the spleen may be absent or multiple, the gut may exhibit malrotation with abnormal mesentery and the lungs may have reversed pulmonary lobulation. Cardiac defects are common and vascular abnormalities may involve the inferior vena cava, portal vein and hepatic artery[2,3]. The autosomal recessive Kartagener's syndrome in human beings, causing 50% situs inversus, is characterised by defective cilia[4].

Although most cases of situs inversus are sporadic, inheritance patterns including X-linked, autosomal recessive and autosomal dominant have been described[5]. Hummel and Chapman first identified the genetic loci involved in localization control in 1959[6]. It was designated the iv gene and has been mapped to chromosome 12[7]. However, only 50% of mutant homozygous mice have situs inversus. This suggests that the normal allele at the iv locus specifies normal symmetry, whereas its absence allows random visceral orientations. Another new inv gene, the second gene found to control lateralization, has been described by Yokoyama and his associates[8].

Patients with situs inversus may be completely asymptomatic, but more commonly, serious cardiac defects preclude long term survival[2]. Although this anomaly is not considered to be a premalignant condition, 16 cases of cancer associated with situs inversus totalis have been reported[9]. It is interesting to note that two such cases were primary liver cancer and both patients underwent left hepatectomy (which is equal to right hepatectomy in the usual cases)[3,10].

From the surgical point of view, recent technologic advances have enabled an accurate diagnosis of situs inversus, unless the patient requires emergency surgical treatment and no time can be spent in ultrasonic or radiologic investigations[10]. Once the diagnosis is made, careful preoperative anatomic assessment is needed since situs inversus is frequently accompanied by other intra-abdominal anomalies. The diagnosis of malignancy associated with situs inversus becomes straight forward once the anatomical transposition of viscerae is appreciated.

The present case report is interesting. This is the third reported case of liver cancer associated with situs inversus totalis. As the patient had liver cirrhosis, the authors decided to resect the postero-superior segment of the liver so as to leave behind as much functioning liver tissues as possible. The anatomical blood supply of the liver had been studied on three liver specimens obtained from bodies in situs inversus. The study showed that anatomical resection of the individual liver segments, like in normal patients, is possible in situs inversus[11]. The authors have now carried out the resection of a single liver segment and confirmed that this can really be done.

W.Y. Lau
A.K.C. Li
Department of Surgery
The Chinese University of Hong Kong
Prince of Wales Hospital
Shatin, New Territories
Hong Kong

### REFERENCES

1. Breuckner M, McGrath J, D'Eustachio P. (1991) Establishment of left-right asymmetry in vertebrates: genetically distinct steps are involved. In: Bock G.R, Marsh J, eds. Biological Asymmetry and Handedness. Chichester, UK: Wiley, 202–19.
2. Fishman L.N, Lavine J.E. (1994) Comments on what's wrong when it 'isn't right: situs inversus and genetic control of organ position. *Hepatology,* **19**: 257–8.
3. Kim YI, Tada I, Kuwabara A, Kobayashi M. (1989) Double cancer of the liver and stomach with situs inversus totalis–*A case report. Jpn J Surg,* **19**: 756–9.
4. Jones K.L. (1988) Smith's recognizable patterns of human malformation. Fourth edition. Philadelphia: W.B. *Saunders.* 543–5.
5. Mathias R.S, Lacro R.V, Jones K.L. (1987) X-linked laterality sequence: situs inversus, complex cardiac defects, splenic defects. *Am J Med Genet,* **28**: 111–6.
6. Hummel K.P, Chapman D.B. (1959) Visceral inversion and associated anomalies in the mouse. *J Hered,* **50**: 10–3.
7. Hanzlik AJ, Binder M, Layton WM, et al. (1990) The murine situs inversus viscerum (iv) gene responsible for visceral symmetry is linked tightly to the lgh-c cluster on chromosome 12. *Genomics,* **7**: 389–93.
8. Yokoyama T, Copeland N.G, Jenkins N.A, (1993) Montgomery C.A, Elder V.B, Overbeak P.A. Reversal of left-right asymmetry: a situs inversus mutation. *Science,* **260**: 679–82.
9. Abdel-Dayem H.M, Motawi S, Jahan S, Kubasik H. (1984) Liver metastasis from embryonal carcinoma of the ovary with complete situs inversus. First reported case and review of the literature. *Clin Nucl Med,* **9**: 558–60.
10. Kanematsu T, Matsumata T, Kohno H, Sugimachi K, Inokuchi K. Hepatocellular carcinoma with situs inversus. *Cancer,* **51**: 549–52.
11. Ion A, Tiberiu C.G. (1982) Anatomical features of liver in situs inversus. *Acta Anat,* **112**: 353–64.

